# Effect of an Augmented Reality Ultrasound Trainer App on the Motor Skills Needed for a Kidney Ultrasound: Prospective Trial

**DOI:** 10.2196/12713

**Published:** 2019-05-01

**Authors:** Florian Ebner, Amelie De Gregorio, Fabienne Schochter, Inga Bekes, Wolfgang Janni, Krisztian Lato

**Affiliations:** 1 Helios Amper Klinikum Dachau Germany; 2 University of Ulm Ulm Germany; 3 Obstetrics and Gynecology Department University of Ulm Ulm Germany

**Keywords:** ultrasound trainer, mobile device, mobile apps, augmented reality, kidney, sensitivity and specificity, ultrasonography, education, simulation training, telemedicine

## Abstract

**Background:**

Medical education is evolving from "learning by doing" to simulation-based hands-on tutorials.

**Objective:**

The aim of this prospective 2-armed study was to evaluate a newly developed augmented reality ultrasound app and its effect on educational training and diagnostic accuracy.

**Methods:**

We recruited 66 medical students and, using imaging and measuring a kidney as quality indicators, tested them on the time they needed for these tasks. Both groups used textbooks as preparation; in addition, the study group had access to a virtual ultrasound simulation app for mobile devices.

**Results:**

There was no significant difference between the study arms regarding age (*P*=.97), sex (*P*=.14), and previous ultrasound experience (*P*=.66). The time needed to complete the kidney measurements also did not differ significantly (*P*=.26). However, the results of the longitudinal kidney measurements differed significantly between the study and control groups, with larger, more realistic values in the study group (right kidney: study group median 105.3 mm, range 86.1-127.1 mm, control group median 92 mm, range 50.4-112.2 mm; *P*<.001; left kidney: study group median 100.3 mm, range 81.7-118.6 mm, control group median 85.3 mm, range 48.3-113.4 mm; *P*<.001). Furthermore, whereas all students of the study group obtained valid measurements, students of the control group did not obtain valid measurements of 1 or both kidneys in 7 cases.

**Conclusions:**

The newly developed augmented reality ultrasound simulator mobile app provides a useful add-on for ultrasound education and training. Our results indicate that medical students’ use of the mobile app for training purposes improved the quality of kidney measurements.

## Introduction

### Background

Sonography is a well-established diagnostic tool and is sometimes used for small interventions. It is a noninvasive treatment or diagnostic tool, is cost effective, has no side effects, and is clinically valuable in nearly all medical disciplines. Technical developments in recent years mean that the examiner requires more skill and knowledge in using ultrasound [1]. As a result, the demand for educational lectures and courses has increased [2-4]. Traditionally, medical journals, hands-on tutorials, and theoretical lectures have been used for keeping doctors up-to-date. One of the difficulties of sonography compared with other imaging technologies is the complex motor hand-eye coordination required. Students are commonly trained in coordination on healthy volunteers with limitations in time and availability. Malignancies or abnormalities are most likely not present in healthy volunteers. Therefore, various models for simulation have been developed [5]. The expense of such simulators, unfortunately, limits their availability for practice.

Due to the technical advances in mobile phones and the common acceptance of augmented reality (AR)—mainly due to the popularity of the video game Pokémon Go [6]—new training possibilities via smartphone have opened up [7]. AR is commonly defined as extended information on a real-world image, compared with virtual reality (VR), which is completely separated from the real-world image. With AR it is now possible to simulate a patient on a smartphone and imitate a sonographic examination.

### Objective

The aim of this cohort study was to determine whether there was a difference in hand-eye coordination and motor skills needed for ultrasound examination between 2 groups of medical students with and without exposure to a VR ultrasound training app for the time and measurements of a kidney ultrasound.

## Methods

### Participants and Procedure

Using the Consolidated Standards of Reporting Trials (CONSORT) and Standards for Reporting of Diagnostic Accuracy Studies (STARD) statements as guidelines, we designed this cohort study, called Ultraschall App Study (UPPS), to evaluate a newly developed ultrasound AR simulator mobile app on its educational and diagnostic effect on 2 cohorts of medical students. The curriculum is an annual schedule resulting in same-year students attending a summer and a winter semester.

We recruited 66 medical students and split them into 2 groups. We determined the starting group (the control group) by flipping a coin. We recruited the control group in the summer term between April and June 2016. We recruited the study group between August 2016 and November 2016 (no student courses are offered in June and July). Participation in the study was offered during a mandatory weekly course in obstetrics and gynecology sonography but participation was voluntary. The lecturer was the same over the recruitment period. No student declined.

**Figure 1:**
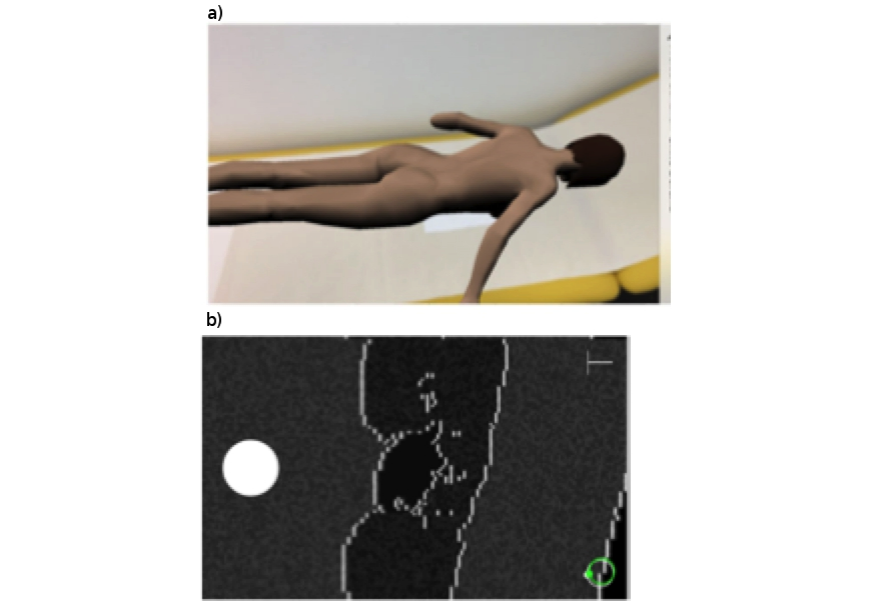
(a) The virtual patient and (b) the kidney scan as shown on the mobile device. The white spot documents the image, T indicates which plane needs documenting, and the green circle indicates the correct position. If the image is off, the circle is red, thus providing immediate feedback to the user. See also a video of the app in Multimedia Appendix 1.

Initially a questionnaire was handed out and participants self-estimated their ultrasound experience (self-estimation was scored on a scale from 0 to 10, with 0 indicating no sonographic experience and 10 indicating a very experienced student). A tutor explained the aim of the following 60-minute study time, and participants were provided with theoretical knowledge for self-study (*Sono-Grundkurs* [8]). The participants were told to aim to visualize and document the reference or tutor kidneys with an ultrasound at the end of this lecture. Study group students additionally had access to the iOS-based AR ultrasound simulator app installed on 3 handheld devices. The study app was designed using the mobile device’s gyroscope to simulate the motion of an ultrasound transducer and was provided in the native language (German). The text files were also translated into English, Hungarian, Romanian, Italian, and Polish by native-speaking colleagues. With the app, training ultrasound motor skills does not need a proper ultrasound machine, nor a patient. It is also independent of time and location, as the mobile device needed is a smartphone or a tablet. Figure 1 shows the virtual patient as displayed on the tracker pattern and the ultrasound mode once the mobile device is close to the virtual skin, showing a kidney scan simulation (Multimedia Appendix 1). One patient was simulated for this proof-of-concept study.

After 60 minutes of self-study in a group, the participants had a brief tutorial on the use of the ultrasound machine (GE Voluson Expert 8, General Electric, GE Medical Systems, Solingen, Germany) set to kidney scan. Then the participants were asked one by one to scan and measure both kidneys of the tutor as accurately as possible and document their scan with the normal images. Starting time was the beginning of the examination, and finishing time was the time stamp on the last picture. We used this time frame to compare the 2 groups and as an internal quality control for the self-estimation. After students finished the documentation, they were given a written multiple choice test (range 0-6 points) to evaluate their theoretical knowledge. Finally, the study group was asked to assess the AR mobile app on a scale from 0 to 10 regarding the usefulness of the app, their recommendation regarding its use, and problems they encountered (responses: yes, no, not yet).

Prior to the study recruitment, we consulted the University of Ulm ethics committee, which exempted the study from ethical approval.

### Statistical Analysis

For the statistical analysis, we used IBM SPSS Statistics for Windows, version 21.0 (IBM Corporation). The descriptive statistic used likelihood tables with absolute and relative likelihood for nominal data and with median and area for ordinal-scaled and metric data.

Due to the significant difference in the distribution of the multiple metric variables (kidney measurements, examination time, age, semester, ultrasound experience, multiple choice test results, and app rating) from the norm (Shapiro-Wilk test), we used exclusive nonparametric statistical analysis. We compared the groups for nominal-scaled (categorial) data or rates with chi-square tests (Fisher exact test; variable: successful visualization of the kidney). We applied Mann-Whitney *U* test to test the differences between 2 independent groups referring to ordinal-scaled or metric data (kidney measurements, examination time, age, semester, ultrasound experience, and multiple choice test results).

We used boxplots for ordinal-scaled and metric data for intergroup visualization (kidney measurements, examination time, age, semester, ultrasound experience, and multiple choice test results). In these plots, the horizontal line is the median and the box symbolizes 50% of the data (interquartile area). The whiskers of the box and whisker plots had a maximum length of 1.5 times the interquartile area. If all data are within these borders, then the minimum and maximum value determine the length of the whisker. All values outside the whiskers are marked as dots. We calculated correlations for ordinal-scaled and metric data according to Spearman rho (ρ). All *P* values are 2-tailed and *P*<.05 was considered significant.

## Results

### Participant Characteristics

A total of 66 medical students participated in our study; 33 students were assigned to the control group and 33 to the study (app) group. There was no significant difference in the parameters age, dominant hand, and sex between the 2 groups (Table 1). Because we recruited participants in the summer/winter term, members of the study group were on average in their ninth semester (range eighth to 12th semester) and the control group were in their eighth semester (range seventh to 10th semester). Prior ultrasound knowledge was similar (study group: median score 2, range 0-4.5; control group: median score 2, range 0-4; *P*=.66). In the study group, more students self-reported AR experience (7/33, 21% vs 1/33, 3%; *P*=.05; Table 1) than in the control group.

### Group Result Comparisons

**Figure 2:**
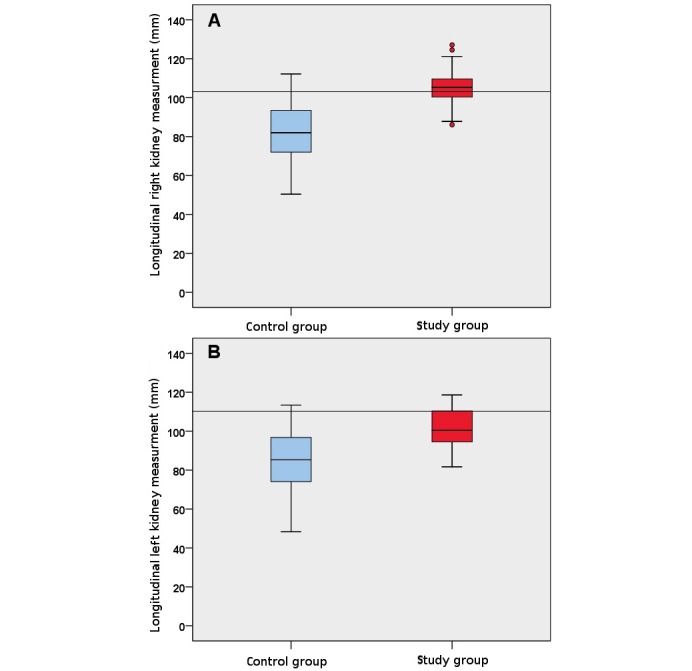
Box and whisker plots comparing longitudinal (a) right kidney and (b) left kidney measurements (in millimeters). The reference kidney is marked at 101 mm (right kidney) and 110 mm (left kidney).

**Figure 3:**
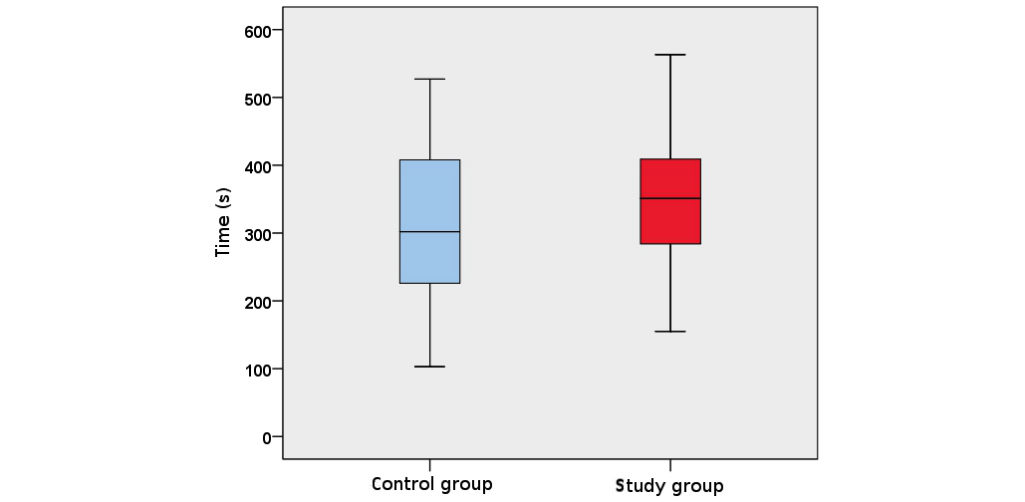
Box and whisker plot of the time to document both kidneys (in seconds).

The study group visualized the kidneys in all cases on both sides, whereas the control group did document the kidney in 7 cases (1 right and 6 left). This resulted in a significant difference for the left kidney (Fisher exact test, *P*=.02). Additionally, the measurement of the kidney length was significantly different (right kidney: study group median 105.3 mm, range 86.1-127.1 mm, control group median 92 mm, range 50.4-112.2 mm; *P*<.001; left kidney: study group median 100.3 mm, range 81.7-118.6 mm, control group median 85.3 mm, range 48.3-113.4 mm; *P*<.001; Figure 2). The measuring time period (in seconds) was not significantly different (study group median 351 s, range 155-563 s, control group median 302 s, range 103-527 s; *P*=.26; Figure 3). There was an inverse correlation between the time needed for kidney documentation and self-reported ultrasound experience (ρ=–.28, *P*=.04). The results of the multiple choice questionnaire were not significantly different between the 2 groups (*P*=.13).

**Table 1:** Comparison of the variables of the 2 groups.

Variable		Control group (n=33)	Study group (n=33)	*P* value
**Age (years)**				.97^a^
	Median	24	24	
	Range	22-31	22-30	
**Sex, n (%)**				.14^b^
	Male	13 (39)	19 (58)	
	Female	20 (61)	14 (42)	
**Dominant hand, n (%)**				.24^c^
	Right	33 (100)	30 (91)	
	Left	0 (0)	3 (9)	
**Self-reported experience with augmented reality, n (%)**				.05^c^
	Yes	1 (3)	7 (21)	
	No	32 (97)	26 (79)	
Theoretical multiple choice test, mean score		36.8	30.2	.18^d^
**Ultrasound experience score (scale 0-10)**				.66^a^
	Median	2	2	
	Range	0-4	0-4.5	

^a^Mann-Whitney *U* test.

^b^Chi-square test.

^c^Fisher exact test.

^d^Asymptotic significance (2-tailed) of the Mann-Whitney *U* test.

## Discussion

### Principal Findings

The transition from reality to virtuality has been described as a reality-virtuality continuum by Milgram et al [9]. The amount of additional virtual information varies depending on the needs and what it is being used for. In AR, additional information is displayed or initiated with barcodes, imaging recognition software, or trackers to enhance reality. This is in contrast to VR, where everything is computer generated.

Computer games and smartphones have helped bring VR and AR into daily life. Google Glass, a brand of smart glasses, received mixed responses when first announced [10,11]. Pokémon Go, introduced in the summer of 2016, could be considered the AR breakthrough. Since then, the usability of AR and VR have continuously improved [12]. The “Pokémon Go effect” may have played a role in our study, as the questionnaires showed an increase in self-reported AR knowledge in the study population (from 3% in April to 21% in September) [6].

Over the last 20 years, several generations of medical apps have been produced. Whereas the first generation were expensive and had potential clinical uses [13], improvements in chip and smartphone technology enabled new possibilities for education and learning using the advances made in the entertainment industry and implementing them in medical education [14]. AR can enhance the learning curve for ultrasound education in combination with theoretical knowledge and motor skills. To date, to our knowledge no smartphone app that can simulate an ultrasound examination has been developed.

To objectively evaluate the effectiveness of the mobile app, we targeted medical students with next to no ultrasound experience but with knowledge of anatomy. The weekly obstetrics and gynecology introductory course proved ideal. This introductory course is mandatory for fourth-year medical students before they are exposed to clinical work. Students tend to communicate with each other about their clinical courses and the examinations at the end. To ensure that the 2 groups in the study would be independent, with minimal exchange of information about the study, we spread the recruitment time over 2 university teaching periods (summer and winter semesters), with 1 semester per group (control and study). Starting with the control group also coincidentally ensured that the following students were not biased by earlier participants. Students also could not find the app in the online store as a training opportunity outside the study, which would have introduced further bias. The tutor was approached by 2 control students once word about the app had spread among the students. We chose the kidney due to its superficial position, homogeneous size (with the normal adult kidney being 100-120 mm [8]), and importance in various disciplines. Little time is needed to learn to do a kidney ultrasound. Our study group’s measurements were closer to the values of the reference kidneys within an hour of practice and significantly different from the general visualization of the kidney.

In a prospective randomized trial, Celebi et al showed similar teaching effectiveness for student tutors and ultrasound experts [3], so our aim was to provide a first evaluation of the effectiveness of a mobile app without a tutor’s supervision. The results add to the observations of Celebi et al and others by showing positive effects after 60 minutes of autonomous practicing [15-17]. As opposed to Celebi et al [3] and Ritter et al [16], our study focused on practicing motor skills by using a smartphone or tablet.

Furthermore, our study included a practical test by visualization of the kidney and a multiple choice questionnaire. Despite the published possibilities of combining practical evaluation methods for teaching interventions, such a practical test is not commonly used for evaluation in a clinical course [17] but, from our point of view, is an essential step for a successful clinical lecture. The significant differences between the control and intervention group in the visualization and measurements show the need for such a hands-on approach. A tutorial including hands-on practice prepares students better for clinical routine, even without further tutor supervision.

### Limitations

Despite these positive results, we identified the following points that need to be addressed. Recruitment bias can only be minimized, as random allocation for each participant is not possible in our setting. The students were assigned on a weekly schedule to a group, and interaction between the groups was known to occur. To reduce this bias, the study protocol grouped the participants per semester. Unfortunately during the study period, the Pokémon Go game became available and VR bias might have had an effect on the results [6]. Students with more VR experience may have better motor skills with their mobile devices due to additional training. As there was no difference in scan time or app rating before and after the release of Pokémon, such a bias seems unlikely. But, with the expected increased use of AR mobile apps, this effect might influence future studies, as a VR-naive comparable control group would be impossible to recruit. On the other hand, the kidney measurements differed between the 2 subgroups and this study, which was not designed to differentiate between preexisting motor skills and app-trained skills. This needs to be evaluated with either a larger number of students or a baseline question regarding the participants’ gaming habits.

The 2-armed study protocol could be further criticized. There is, to our knowledge, no evidence-based statement for medical education trials, so we wrote the study protocol with the CONSORT and STARD statements as guidelines. The mobile app was designed to enhance the learning experience with a textbook by enabling the student to practice his or her motor skills and experience the theoretical facts on screen. This is in line with the results of the Extended Focused Assessment with Sonography for Trauma (eFAST) study [18], which showed no benefit for mobile e-learning compared with traditional learning. eFAST focused on the difference in theoretical learning and not on the motor skills as in our trial. The cost of developing such an app can be criticized. As the trial version of this app is available at no cost, we disagree with Nilsson et al [18] and do see a cost effectiveness for motor training, especially because the time of tutors, costs for ultrasound machines, and secondary costs (eg, room, missed outpatient clinic) are minimized with this app and no other cost-effective motor skills training method is available. Also, after app training, time is saved by improved imaging and, ultimately, diagnosis in the clinical setting. Besides those savings, the app development was the biggest cost factor. With only 1 organ and only 1 individual (eg, no variation in subcutaneous fat tissue) in the app, the costs surely outweigh the benefits per user, but there is the potential to simulate more difficult clinical cases such as obese patients, cardiac scans, or fetal organ screening in future studies.

We could have applied the Objective Structured Assessment of Ultrasound Skills criteria like Tolsgaard et al [19] applied them for a structured examination of the lung. Here the ultrasound federations could help future studies by providing variables. These proposed benchmarks based on current teaching models provide an expert’s feedback on imaging quality. A mobile app could support the expert by guiding the user to the “ideal” image, ultimately providing rapid feedback and improving image quality beyond the current expectations regarding time and practice. This approach adapts individual differences in the learning curve [4,20] by being independent of expertise, time, location, and place. This freedom could be appealing to a wide range of students, and our results also show no sex difference in the acceptance of the app. With this home-based learning, the app could be used to prepare participants prior to an ultrasound course in order to maximize the learning effect.

### Conclusion

We found that students can be trained in the motor skills needed for ultrasound examination using an AR app. Within a short training period, participants documented the kidney significantly better. The main advantage of the app is the freedom to train without a patient and a real ultrasound machine. With the implementation of immediate feedback on imaging quality and various scenarios and patients, such apps could be a valuable enhancement of lectures, courses, and textbook-based learning. This should result in more effective learning and improved clinical skills. Further benefits include the freedom to train in terms of time, model or patient, and place at a reasonable cost.

## Appendix

Multimedia Appendix 1 Video of the training app.

## Abbreviations

AR: augmented reality
CONSORT: Consolidated Standards of Reporting Trials
eFAST: Extended Focused Assessment with Sonography for Trauma
STARD: Standards for Reporting of Diagnostic Accuracy Studies
UPPS: Ultraschall App Study
VR: virtual reality
